# Co-application of biochars and *Piriformospora indica* improved the quality of coastal saline soil and promoted the growth of forage

**DOI:** 10.3389/fpls.2024.1434097

**Published:** 2024-08-12

**Authors:** Qicong Wu, Ke Ning, Bingqian Liu, Xuejia Zheng, Chen Li, Xin Li, Xiaohu Zhou, Jiawang Li, Jiajing Li, Congzhi Zhang, Zhi Dong

**Affiliations:** ^1^ Co-Innovation Center for Soil-Water and Forest-Grass Ecological Conservation in Yellow River Basin of Shandong Higher Education Institutions, College of Forestry, Shandong Agricultural University, Tai’an, China; ^2^ Mountain Tai Forest Ecosystem Research Station of State Forestry and Grassland Administration, Tai’an, China; ^3^ Yantai Muping District Agricultural Technology Promotion Center, Yantai, China; ^4^ State Experimental Station of Agro-ecosystem in Fengqiu, State Key Laboratory of Soil and Sustainable Agriculture, Institute of Soil Science, Chinese Academy of Sciences, Nanjing, China

**Keywords:** saline-alkali soil, *Piriformospora indica*, biochar, photosynthesis, forage

## Abstract

Soil quality is defined as the ability of soil to maintain the soil environment and the biosphere. Due to the limitation of salt and alkali stress, soil quality can be reduced, which in turn affects agricultural production. Biochar is widely used in saline–alkali land improvement because of its special pore structure and strong ion exchange ability, while *Piriformospora indica* is widely used in saline–alkali land improvement because it can symbiose with plants and improve plant stress resistance. However, the synergistic effect of combined biochar application and inoculation of *P. indica* on the quality of saline–alkali soil and plant development is uncertain. Hence, we investigated the combined influences of biochar and *P. indica* on the soil physicochemical characteristics, as well as the growth and chlorophyll florescence of sorghum–sudangrass hybrids (*Sorghum bicolor* × *Sorghum sudane*) in our study. The results indicated that after applying biochar and *P. indica* together, there was a considerable drop in soil pH, conductivity, Na^+^, and Cl^−^ concentrations. Meanwhile, the soil organic matter (SOM), available phosphorus (AP), and alkaline hydrolyzable nitrogen (AN) increased by 151.81%, 50.84%, and 103.50%, respectively, when the Bamboo biochar was combined with 120 ml/pot of *P. indica*. Eventually, sorghum–sudangrass hybrid biomass, transpiration rate, and chlorophyll content increased by 111.69%, 204.98%, and 118.54%, respectively. According to our findings, using *P. indica* and biochar together can enhance soil quality and plant growth. The results also provide insights to enhance the quality of saline–alkali soils and the role of microorganisms in nutrient cycling.

## Graphical Abstract

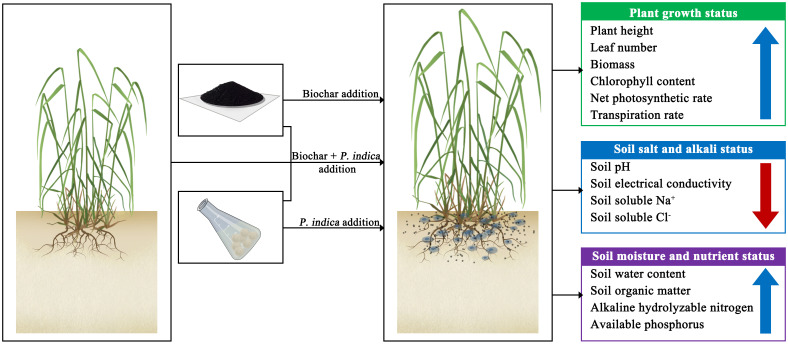

## 1 Introduction

Soil salinization is a widespread problem globally, and the area is approximately 1.1 billion hectares and is increasing by 10% annually (Kumar et al., 2017; Yang et al., 2023). Coastal saline soils are gradually formed from saline silt, and their primary source of salt is seawater. The quality of coastal saline soils is generally poor, and directly prevents photosynthesis and plant development, which is undesirable for the health of plants (Peng et al., 2012). The strategies used to improve saline–alkali soils mainly include physical, biological, and chemical measures. The current approaches used to improve saline–alkali soils involve the use of stable, effective, green, and low-cost composite soil improvement agents.

Biochar has received considerable attention as a highly stable soil amendment in recent years (Haider et al., 2022). Under salinity stress, biochar can enhance soil quality and plant development (Haider et al., 2020; Usman et al., 2016; Zheng et al., 2018; Mi et al., 2023). Biochar has a variety of bioactive compounds, which are conducive to soil nutrient sequestration, in addition to improving soil fertility, nutrient utilization, soil properties, and microbial habitats (Lashari et al., 2015; Chauhan et al., 2024). Biochar can regulate soil organic carbon content and serve as a conditioner for the soil when there is a high salinity stress (Luo et al., 2017; Wu et al., 2020; Mi et al., 2024). It is possible to increase crop productivity and improve soil quality simultaneously. Biochar was recently discovered to alleviate salt and oxidative stress in saline soils, and promote nutrient uptake and phytohormone regulation in plants (Usman et al., 2016). Although biochar has numerous advantages over other amendments, variations have been observed in the physicochemical properties (Uzoma et al., 2011) and adsorption characteristics (Luo et al., 2017) of biochars derived from various materials due to the different raw materials used to prepare biochar. High temperature-produced biochar is linked to poor soil nutrient levels, and they limit soil nutrient improvement to a certain extent (Yang et al., 2021). It has been demonstrated that the poor nutrient content of biochar can be efficiently improved by applying chemical fertilizers, such as both organic and inorganic fertilizers, in conjunction with biochar. Nevertheless, the application of chemical fertilizers is often associated with secondary pollution. Microorganisms and biofertilizers derived from them have the advantages of low pollution and high efficiency when compared to conventional chemical fertilizers and have been gradually applied to improve agricultural production. Soil pH tends to decrease after the application of microorganisms (Anam et al., 2019). Microorganism application is proven to substantially boost SOM content and improve soil quality. The parameters to consider include characteristics such as water retention capacity and permeability (Jabborova et al., 2022).

*Piriformospora indica* (*P. indica*, Sebacinaceae) is a plant root endophytic fungus that has great potential for biocontrol against abiotic stress and soil improvement, and its biological effects and mechanism of action are currently subjects of interest among researchers (Gill et al., 2016). According to a previous study, *P. indica* can colonize the roots (Johnson et al., 2014) and raise plant tolerance to abiotic stress by promoting mineral uptake (Xu et al., 2018) inducing plant resistance (Hussin et al., 2017; Karimi et al., 2022). However, the efficiency of microbial remediation may be hindered by nutrient limitation, lack of sustained release, and lack of habitats. In this regard, biochar has positive attributes due to its porous structure, which promotes microbial growth and improves soil quality (Videgain et al., 2021). We hypothesize that the limitations can be overcome by integrating microbes and biochar in soil remediation. In summary, *P. indica* and biochar have a high potential to improve soil quality; however, comprehensive impacts on soil between *P. indica* and biochar have only been studied in a few studies, and the underlying mechanisms are yet unknown.

Therefore, this study investigated the influences of biochar and *P. indica* on coastal saline soils and the growth of sorghum–sudangrass hybrids. The research aimed to address the following research questions: (1) whether biochar with *P. indica* reduces soil salinity and alkalinity; (2) whether there is any effect of biochar and *P. indica* in improving soil water holding capacity, nutrients, and soil quality; (3) whether biochar and *P. indica* increases growth and photosynthesis of sorghum–sudangrass hybrids. The results could provide valuable information for the sustainable utilization of coastal saline soils and enhance our understanding of biochar–microbial interactions. The findings could be useful for the long-term use of coastal saline soils, as well as for better understanding of biochar–microbial interactions.

## 2 Materials and methods

### 2.1 Preparation of endophytic fungi, biochar, and soil

The fungal strain *P. indica* was cultured as described by Liu et al. (2022). For 1 week at 30°C, the fungus was inoculated on potato dextrose agar (PDA) and then activated for a week at the same temperature on a brand-new PDA tablet. The fungal culture medium was transferred to a 300-ml conical flask with 200 ml of the liquid medium added to each conical flask. Subsequently, eight small pieces (1 cm^3^) of the conical flask were filled with a solid medium containing mycelia. For 7 days in the dark, the mycelial masses were inoculated in potato dextrose broth and allowed to proliferate at 25°C and 150 r/min. Mycelia, as the inoculum, were transferred to a conical flask, 200 ml of spore suspension was added, and mixed using a stirrer.

The biochar was provided by Lize Environmental Technology Company. Biochar used in this study was derived from corn stover and bamboo biochar obtained via pyrolysis for 3 h at 500°C in a muffle furnace. The characteristics of corn stover-derived biochar are as follows: pH of 9.5, electrical conductivity (EC) of 4.53 mS/cm, soil organic carbon (SOC) content of 42.21%, pore volume of 57.28%, and moisture content of 10%. The properties of bamboo-derived biochar are as follows: pH of 8.5, EC of 1.74 mS/cm, SOC content of 79.00%, pore volume of 58.61%, and moisture content of 10%.

Soil samples were collected from the surface saline–alkali soil layer in Dongying, Shandong Province, China. The chemical properties of soils collected for the pot experiments are as follows: pH of 8.10, soluble Na^+^ was 0.051%, soluble Cl^−^ was 0.089%, soil organic matter (SOM) was 0.91%, alkaline hydrolyzable nitrogen (AN) was 5.6 mg/kg, and available phosphorus (AP) was 14.56 mg/kg. The soil type is saline tidal soil with moderate and mild salt stress.

### 2.2 Experimental design

There were three replicates in the completely randomized design of the study. Three treatments of biochars (corn stover-derived biochar, bamboo-derived biochar, and non-biochar) and four inoculation amounts of *P. indica* (0, 40, 80, and 120 ml/pot) were used for the experiment. Thus, 12 combinations were used in the experimental setup (**Table 1**). The pot experiment started on 25 May 2022 and ended on 9 August 2022; the trial lasted for 78 days.

**Table 1:** All experimental treatments.

Treatments	Types of biochar	Inoculation amounts of *P. indica*
P_0_	Non-biochar (P)	0 ml/pot
P_40_		40 ml/pot
P_80_		80 ml/pot
P_120_		120 ml/pot
CP_0_	Corn stover biochar (CP)	0 ml/pot
CP_40_		40 ml/pot
CP_80_		80 ml/pot
CP_120_		120 ml/pot
BP_0_	Bamboo biochar (BP)	0 ml/pot
BP_40_		40 ml/pot
BP_80_		80 ml/pot
BP_120_		120 ml/pot

### 2.3 Planting preparation and soil sampling

Five seeds of sorghum–sudangrass hybrids were selected, dipped for 24 h in water, and then put in pots with 4 kg of saline–alkali soil and 5% (w/w) biochar. After the seeds were germinated, they were immediately inoculated and cultured by root-filling method, and the liquid of *Piriformospora indica* was broken up evenly. The seedlings were moderately watered during growth to maintain the soil moisture content at 70% of the maximum field water capacity. Management practices, such as light and watering, were consistent in all treatments during the experimental period. Mycorrhizal infection and plant height, number of leaves, and biomass were determined at harvest. The selected soil was air dried naturally, milled into fine particles, besides sifted to determine the soil’s physicochemical properties and nutrient content.

### 2.4 Soil and plant analyses

The dry and fresh weights of the roots and leaves were measured after 10 weeks of treatment. After the plant samples were dried in an oven at 70°C until a constant weight was reached, the dry weight was calculated. The magnified intersection approach was used to measure the extent of mycorrhizal colonization of plant roots. The roots were initially digested in 10% potassium hydroxide (w/v) and then rinsed in water. The amount of chlorophyll in plant leaves was determined by extracting leaf samples (0.1 g) using 95% ethanol. At 663 and 645 nm, the absorbance of total chlorophyll was measured. After three measurements of each sample, the average value was determined.

For each area, three randomly selected plants, two fully developed leaves, as well as associated metrics, which include net rates of photosynthesis, transpiration of the leaves rate, and conductance of stomatal cells, were utilized to assess gas exchange. The measurements were taken 2 months following the planting. Gas exchange measurements were carried out with a portable open-flow gas exchange system (LI-6800; Li-Cor Biosciences, Lincoln, NE, USA) at the top region of every leaf that was exposed to sunlight. Every measurement was conducted with the same treatments: 25°C leaf temperature, 300 μmol/s of flow rate, 400 μmol/mol of CO_2_ concentration inside the leaf chamber, and 50%–60% relative humidity. Following a 5-min acclimatization period, the photosynthetic parameters were measured three times to get stable data for each treatment.

Soil pH and EC were measured using standard pH (soil/water, 1:5) and conductivity meters, respectively. Soil water content was determined gravimetrically after drying the soil samples in an oven at 105°C for 24 h. Soil-soluble Cl^−^ content was measured by titrating with silver nitrate standard solution using potassium chromate as an indicator. Soluble Na^+^ was determined using flame photometry. By applying the alkaline diffusion method, available nitrogen (AN) was ascertained. AP was determined using the molybdenum–antimony anti-spectrophotometric method followed by extraction with sodium hydrogen carbonate solution. The volumetric technique with potassium dichromate was used to calculate SOM.

### 2.5 Statistical analysis

Statistical analyses were performed using SPSS 13.0 (SPSS Inc., Chicago, IL, USA). A two-way analysis of variance was used to determine the effects of *P. indica* and biochar and their interactive effects on soil quality and plant growth promotion. Duncan’s multiple range was used for *post hoc* comparisons. Here, path analysis was used to specifically identify the direct and indirect effects of biochar and *P. indica* on growth and photosynthesis of sorghum–sudangrass hybrids, as well as the soil physicochemical properties and nutrient contents.

## 3 Results

### 3.1 Effect of biochar and *P. indica* on soil properties

**Figure 1:**
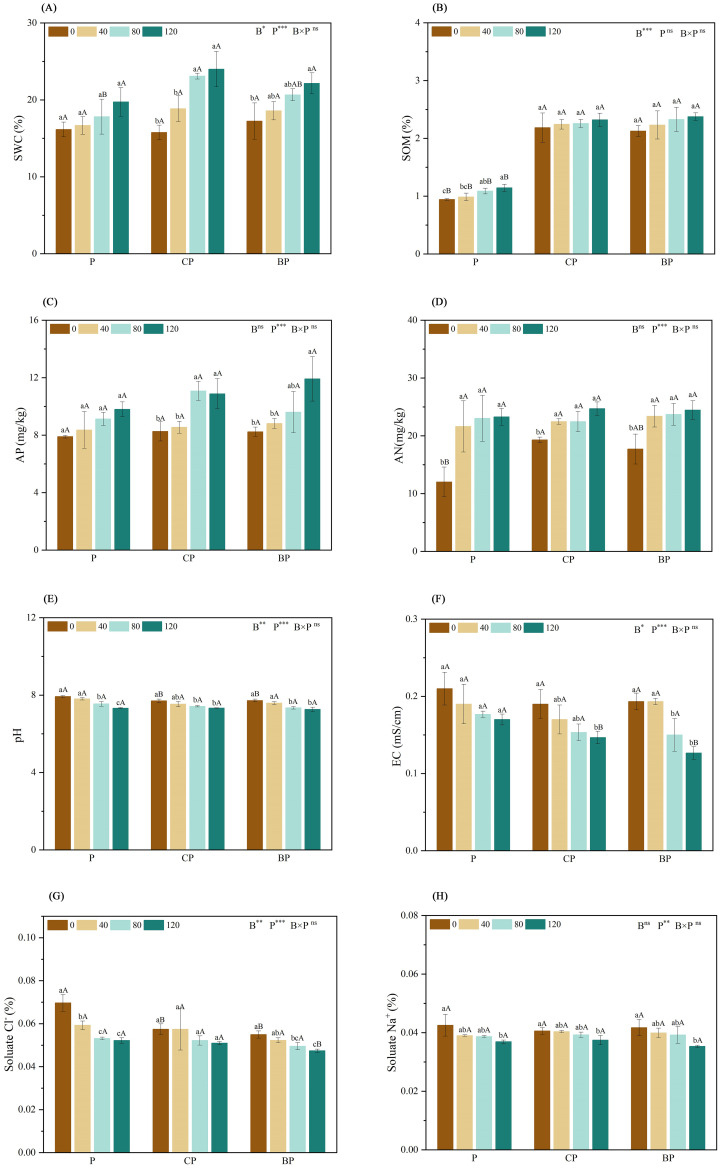
Effect of combined application of biochar and *P. indica* on soil Ph **(A)**, SOM **(B)**, AP **(C)**, AN **(D)**, pH **(E)**, EC **(F)**, soluble Na^+^ **(G)**, and soluble Cl^−^ contents **(H)**. Duncan’s multiple range was used to indicate a significant difference at p < 0.05 for treatments (n = 3). Uppercase letters indicate significant differences between different biochar types at the same *P. indica* dose, whereas lowercase letters indicate significant differences between the same biochar types at different *P. indica* doses. *p < 0.05; **p < 0.01; ***p < 0.001; ns, no statistical significance. SWC, soil water content; SOM, soil organic matter; AP, available phosphorus; AN, alkaline hydrolyzable nitrogen; EC, electrical conductivity.

Soil water contents of CP and BP treatments were the highest at *P. indica* doses of 80 and 120 ml, respectively (**Figure 1A**). Compared with the control, The co-application of *P. indica* and biochar significantly increased SOM by approximately 145.90% (CP120) and 151.81% (BP120). Moreover, while biochar and *P. indica* worked together, the SOM content in the BP treatment slightly exceeded that in the CP treatment (**Figure 1B**). The highest AP contents were observed in the CP and BP treatments (11.08 and 11.92 mg/kg) at *P. indica* doses of 80 and 120 ml/pot, respectively (**Figure 1C**). Inoculation with *P. indica* had a significant effect on AN content (p < 0.001), with AN content increasing slightly with an increase in *P. indica* dosage (**Figure 1D**). Both BP and CP treatments’ AN content did not work apparently by increasing the dosage of *P. indica*.

According to the results, inoculation with *P. indica* effectively decreased soil pH as well as EC, with the effect of *P. indica* increasing with an increase in its dosage. Compared with the control, The co-application of *P. indica* and biochar significantly decreased pH by approximately 7.57% (CP120) and 8.41% (BP120) (**Figure 1E**). The combined addition of biochar and *P. indica* reduced soil EC, and the effect was greater than that of either biochar or *P. indica* alone. Soil EC was the lowest in the BP treatment with *P. indica* doses of 120 ml (**Figure 1F**). Soil-soluble Na^+^ and Cl^−^ contents reduced significantly in the P, CP, and BP treatments (**Figures 1G, H**), with the strongest effect being observed in the treatment with the highest dose of *P. indica*.

### 3.2 Colonization of plant roots by *P. indica*

**Figure 2:**
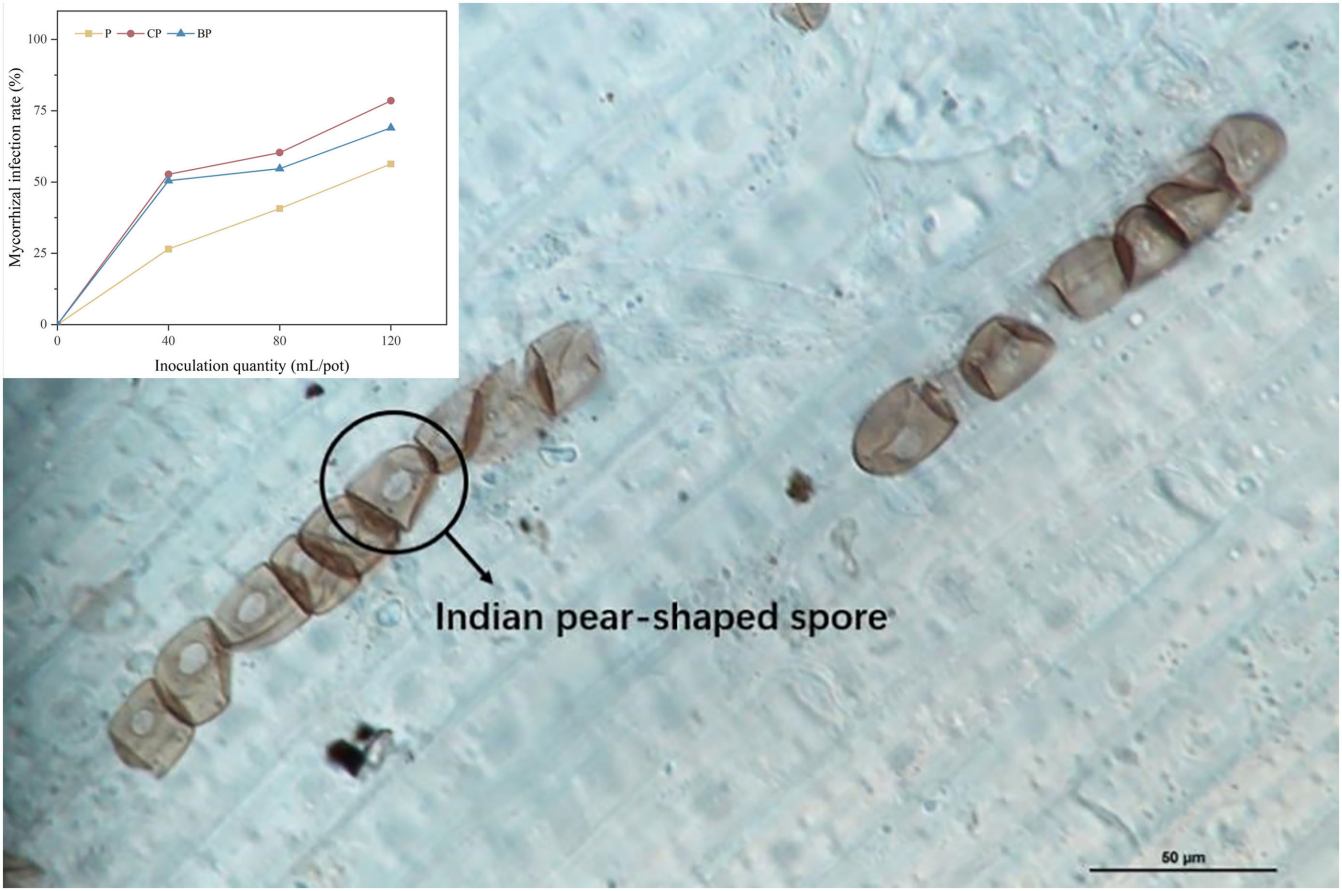
Colonization of sorghum–sudangrass hybrid roots by *P. indica* (magnification, ×100). Colonization rates of *P. indica* in different treatments.

Colonization of the plant root system by *P. indica* is illustrated in **Figure 2**. The highest mycorrhizal colonization was discovered in the CP treatment (52.70%–78.50%), followed by BP (50.45%–69.00%) and P (26.50%–56.33%) treatments (**Figure 2**). *P. indica* colonization of roots was in the order of CP > BP > P. The application of biochar increased *P. indica* colonization of the root system of sorghum–sudangrass hybrids. Colonization of plant roots by *P. indica* was higher in treatments with corn stover-derived biochar than in the treatments with bamboo biochar, and fungal colonization increased with an increase in *P. indica* dosage (**Figure 2**).

### 3.3 Effects of biochar and *P. indica* on sorghum–sudangrass hybrid growth and photosynthetic parameters

**Figure 3:**
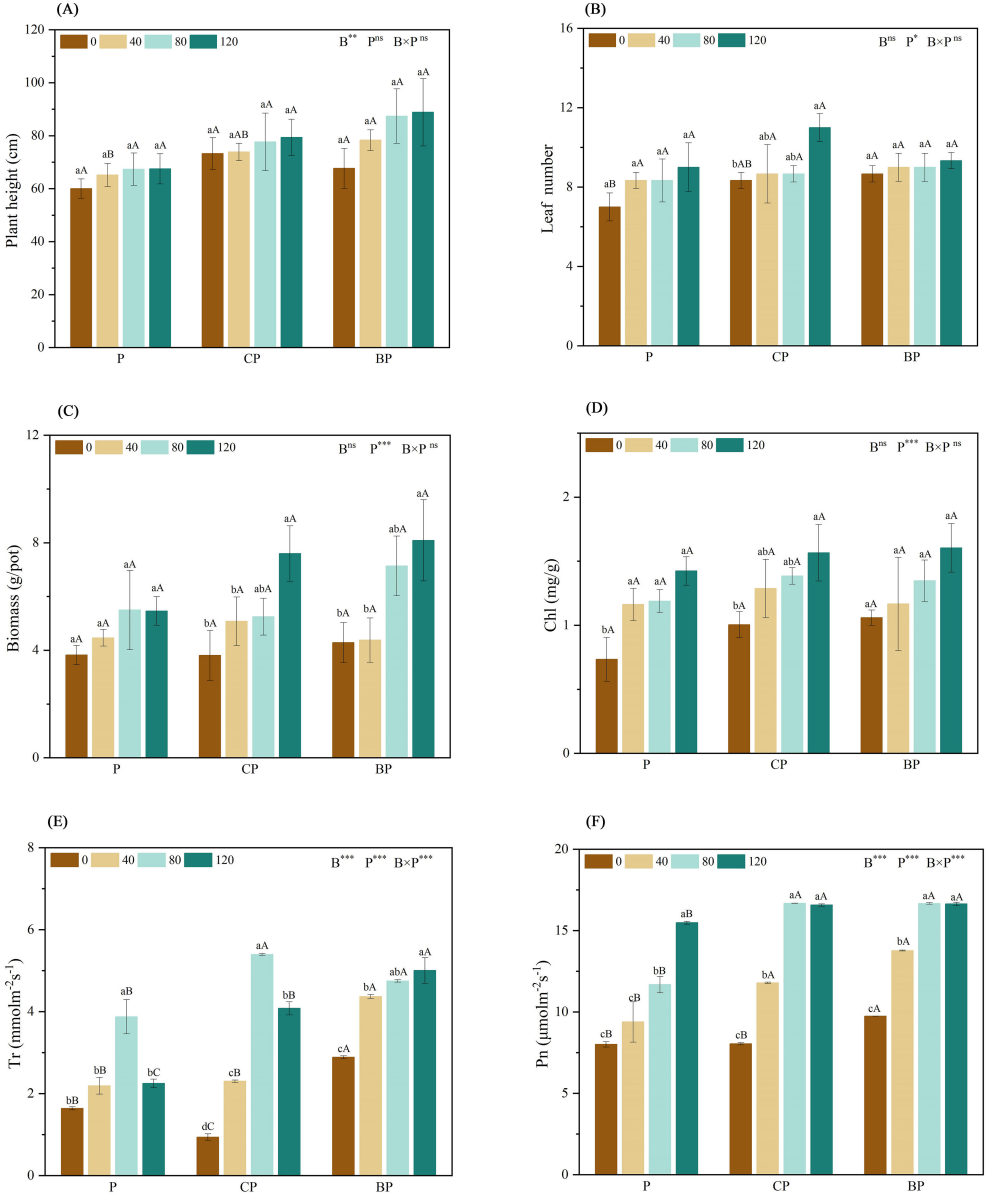
Effect of combined application of biochar and *P. indica* on plant height **(A)**, leaf number **(B)**, biomass **(C)**, Chl **(D)**, Tr **(E)**, and Pn **(F)**. Duncan’s multiple range was used to indicate a significant difference at p < 0.05 for treatments (n = 3). Uppercase letters indicate significant differences between different biochar types at the same *P. indica* dose, whereas lowercase letters indicate significant differences between the same biochar types at different *P. indica* doses. *p < 0.05; **p < 0.01; ***p < 0.001; ns, no statistical significance. Chl, chlorophyll content; Pn, net photosynthetic rate; Tr, transpiration rate.

The biomass of the plants was measured and recorded to determine the tolerance of the hybrids to saline–alkali soils. The combined application of biochar and *P. indica* significantly increased the biomass of sorghum–sudangrass hybrids when compared to that of the control group. Plant growth was higher when biochar and *P. indica* were applied in combination than under single treatments, and plant growth increased with an increase in *P. indica* dose. The combined application of biochar and *P. indica* increased plant height by 32.22% (CP120) and 48.06% (BP120), biomass by 98.87% (CP120) and 111.69% (BP120), and leaf number by 57.14% (CP120) and 33.33% (BP120) when compared to those of the control (**Figures 3A–C**).

The findings indicated that the combined application of biochar and *P. indica* substantially increased chlorophyll content, and net photosynthetic and transpiration rates when compared to either biochar or *P. indica* treatment alone (**Figures 3D–F**). When biochar and *P. indica* were applied together, the net photosynthetic rate of sorghum–sudangrass hybrids increased twofold above the control. The net photosynthetic rate of plants treated with either BP or *P. indica* alone increased gradually when compared to that of the control. Furthermore, when biochar was applied, the net photosynthetic rate reached the highest value when the *P. indica* dosage reached 80 ml/pot. The highest transpiration rate was observed in P and CP treatments at a *P. indica* dose of 80 ml/pot. Conversely, the BP therapy showed the highest transpiration rate at a *P. indica* dose of 120 ml/pot.

### 3.4 Relationships between parameters

**Figure 4:**
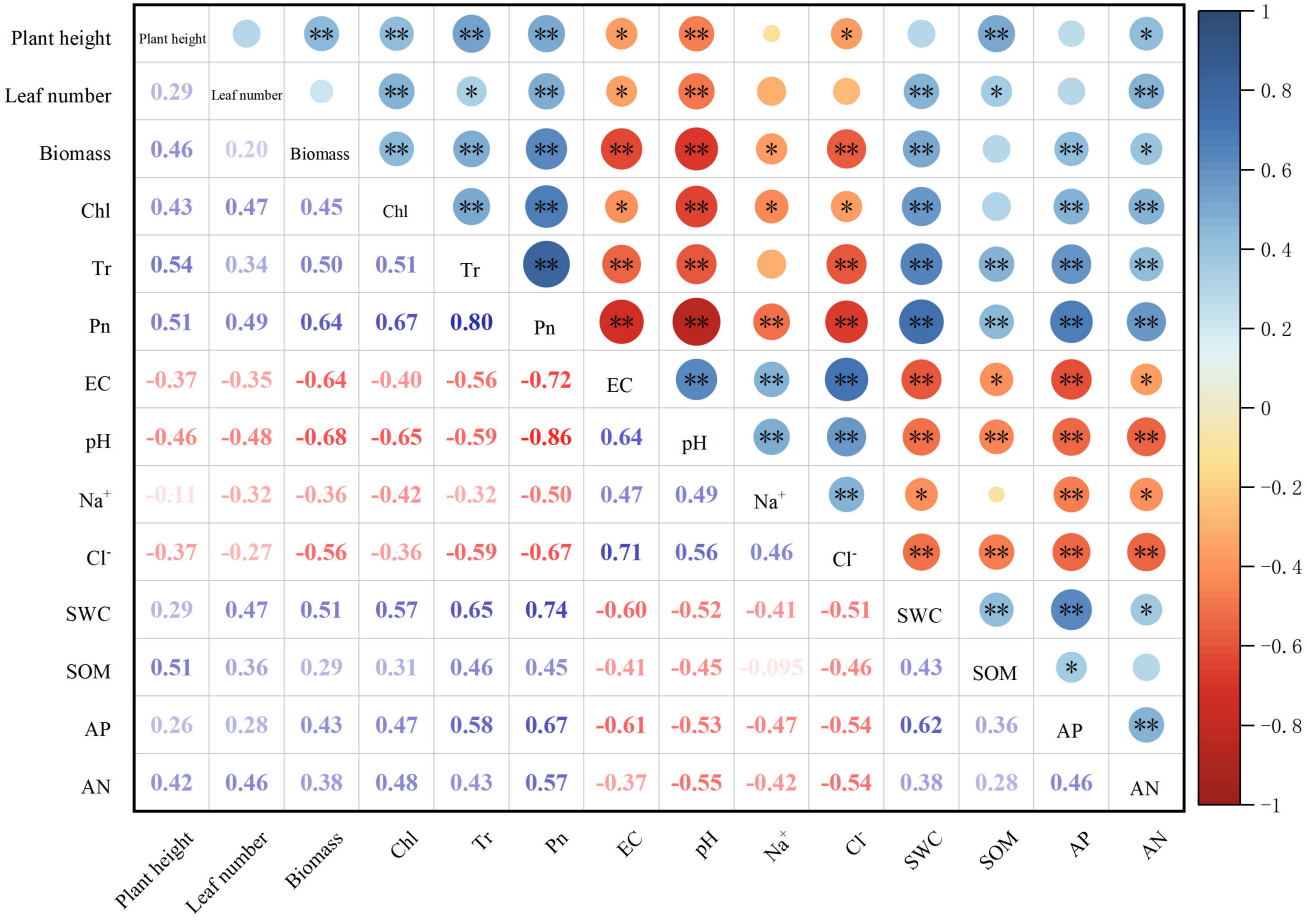
Correlations between soil properties and sorghum–sudangrass hybrids properties. The correlation was evaluated by Pearson correlation coefficient. Red indicates a negative correlation, and blue a positive correlation, and the strength of color reflects the strength of the correlation. *p < 0.05; **p < 0.01.

Correlation analysis revealed that soil pH, EC, Na^+^, and Cl^−^ were negatively correlated with the biomass of sorghum–sudangrass hybrids (**Figure 4**). On the contrary, SWC, AP, and AN were positively correlated with the biomass of sorghum–sudangrass hybrids. Concurrently, chlorophyll content, and net photosynthetic and transpiration rates were positively correlated with the biomass of sorghum–sudangrass hybrids. Compared with the leaf number, the correlation between plant height and biomass was stronger.

### 3.5 Biochar and *P. indica* promote sorghum–sudangrass hybrid growth by improving the soil environment and leaf photosynthesis

**Figure 5:**
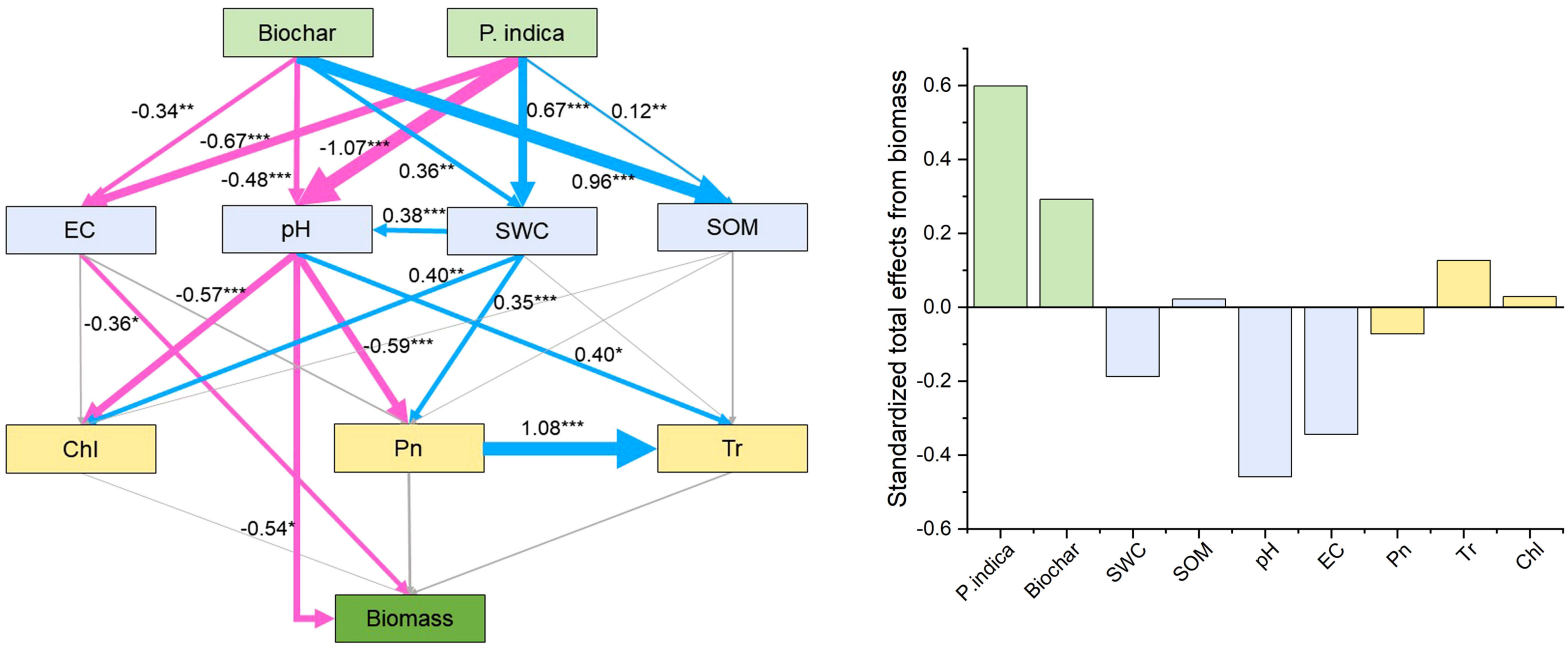
Direct and indirect effects of biochar and *P. indica* on growth and photosynthesis of sorghum–sudangrass hybrids, as well as the soil physicochemical properties and nutrient contents. Models satisfactorily fitted to our data, as suggested by the Chi-square (χ2), goodness of fit index (GFI) and root square mean error of approximation (RMSEA) values [χ2 = 21.114 (p =0.331), GFI = 0.901, RMSEA = 0.056]. Blue lines indicate positive effects, while red lines indicate negative effects. Non-significant effects are indicated by gray lines. The width of the arrows indicates the strength of the significant standardized path effect. *p < 0.05; **p < 0.01; ***p < 0.001. Chl, chlorophyll content; Pn, net photosynthetic rate; Tr, transpiration rate; EC, electrical conductivity; SWC, soil water content; SOM, soil organic matter.

The direct and indirect effects of *P. indica* and biochar on the development and photosynthesis of sorghum–sudangrass hybrids were predicted using path analysis, as well as the physicochemical characteristics and nutrient contents of the soil (**Figure 5**). Path analysis showed that EC and pH had a direct effect on biomass. Biochar, *P. indica*, SOM, Tr, and Chl had positive effects (standardized coefficients = 0.29, 0.60, 0.02, 0.13 and 0.03, respectively), while SWC, pH, EC, and Pn had negative effects on sorghum–sudangrass hybrid growth (standardized coefficients = −0.19, −0.46, −0.34, and −0.07, respectively).

## 4 Discussion

### 4.1 Biochar and *P. indica* reduce soil pH and salt ion concentration

In this study, the combined application of biochar and *P. indica* exhibited a slight impact on pH likely due to the robust buffering capacity of saline soils. The influence of *P. indica* alone on soil pH was more pronounced than that of biochar alone, which might be attributed to biochar’s high ash content enhancing alkalinity (Nguyen et al., 2017). Soil pH is closely associated with plant growth and soil quality, and the enhanced growth of sorghum–sudangrass hybrids could be attributed to low soil pH and EC (Kumar et al., 2017). The combined application of biochar and *P. indica* reduced soil pH, although the reduction in pH was not significant as it cannot entirely explain the improvement in plant growth. Our findings are corroborated by a recent survey that revealed an insignificant relationship between biochar and fungi, as well as soil pH (Ndiate et al., 2021).

Notably, the combined application of biochar and *P. indica* reduced soil EC by 19.05%–34.20%, with the effect of both treatments being stronger than that of either biochar alone, which is consistent with hypothesis 1. The high adsorption capacity of biochar for soil salts can mitigate soil salinity to a certain extent (Haider et al., 2022; Qian et al., 2023). Additionally, the application of biochar to saline soils diminishes salinity by enhancing soil porosity and expediting salt leaching, attributed to biochar’s porous structure (Lashari et al., 2015). The study demonstrates biochar’s capability to reduce high salt concentrations in coastal saline soils evidenced by the decrease in soluble Na^+^ and Cl^−^ contents. For instance, using biochar in corn fields reduced EC, Na^+^, and Cl^−^ concentrations of corn fields by more than 30% (Lashari et al., 2015). Although the application of both biochar and *P. indica* enhanced the salt stress tolerance of *Gordonia* species, no significant synergistic effect was observed between the two in improving soil quality under salinity stress. Based on pertinent parameters, such as stress indicators, *P. indica* exhibited a more pronounced mitigating impact than biochar when subjected to salt stress. Furthermore, sorghum–sudangrass hybrids are pioneer plants adept at tolerating saline soils; hence, the combined application of biochar and *P. indica* could have contributed to the reduction in soil salinity, thereby enhancing the performance of the hybrids.

### 4.2 Biochar and *P. indica* increase soil moisture and nutrient contents

Biochar application can increase soil fertility and the soil’s ability to hold water, which in turn, promotes plant growth (Lashari et al., 2013; Liang et al., 2021; Qian et al., 2023). Considering the study’s observations, the application of biochar with or without *P. indica* increased SOM, AN, and AP contents of the saline–alkali soils, and the effect of the combined application of biochar and *P. indica* was significantly higher than that of either biochar alone (**Figure 1**), which supported hypothesis 2.

The rise in soil moisture and nutrient contents might be linked to the ability of *P. indica* to form a network of mycelium and hyphae around plant roots that extends to the inter-roots (Hammer et al., 2015), which promotes water and nutrient uptake (Ma et al., 2011). Furthermore, the mycelial network within the inter-root soil stabilizes soil aggregates, which in turn, enhanced the soil water holding capacity (Jisha et al., 2019). A significant increase in SOM content was observed following the application of biochar proposing an increase in soil nutrients that, in turn, enhanced plant growth. The results could be associated with the high carbon content of biochar (Kamau et al., 2019) and the promotion of effective phosphorus utilization by biochar (Bornø et al., 2018). Besides, the application of *P. indica* stimulates the production of some functional enzymes in plants (Sahu et al., 2019), such as phosphatase, which effectively promotes nutrient cycling, accelerates the conversion of ineffective phosphorus in the soil (Veresoglou et al., 2012; Babu et al., 2014). In this study, the nutrient content of soil amended with a combination of biochar and *P. indica* was higher than that of soil amended with biochar alone, which demonstrates the role of biochar and *P. indica* in improving nutrient utilization (Li et al., 2022a; Li et al., 2022b). Furthermore, *P. indica* can increase the number of microorganisms in saline soils, improve the soil microbial community structure and the soil environment, and enhance soil fertility (Mukherjee et al., 2021). The results demonstrated that using *P. indica* and biochar together increased the nutrient content of coastal saline soils. However, no discernible synergistic benefit was observed after the application of *P. indica* and biochar on soil nutrients in this study suggesting that various mechanisms underlie the effect of both amendments on plant yield. Consequently, further studies on plant physiology, microbial communities, and soil parameters are required.

### 4.3 Biochar and *P. indica* promote sorghum–sudangrass hybrid growth by improving the rhizosphere micro-ecological environment and photosynthetic performance

In the current study, the application of biochar, either independently or in conjunction with *P. indica*, considerably increased the biomass, height, and quantity of leaves of sorghum–sudangrass hybrids, which corresponds with the results of an earlier investigation (Kamau et al., 2019). The combined application of biochar and *P. indica* promoted plant growth, biomass accumulation, and seed germination when compared to the application of biochar alone. The highest plant biomass was recorded in the BP120 treatment (**Figure 3**). The findings corroborate hypothesis 3 and demonstrate that biochar and *P. indica* application are effective strategies for facilitating the growth of plants. Ndiate et al. (2022) observed that the integrated use of biochar and fungi markedly elevated root fresh weight (24.9%), shoot fresh weight (75.7%), and plant height (14.1%), and photosynthetic pigment production (30.2%–54.8%) of wheat when stressed by salinity.

The results of correlation analysis and path analysis showed that biochar and *P. indica* increased sorghum–sudangrass hybrids biomass mainly by reducing EC and pH and increasing SOM, Tr, and Chl (**Figures 4**, **5**). Biochar enhanced root colonization by *P. indica* by promoting colonization of beneficial microorganisms in the inter-root soil and improving the survival of fungi (Ferreiro and Fu, 2016). Concurrently, the addition of biochar promoted the growth and metabolism of superior salt-tolerant bacteria further improved plant stress resistance and promoted plant growth (Wang et al., 2024). *P. indica* contains soil activators that increase soil fertility, quicken the decomposition of organic matter, and encourage the uptake of nutrients and plant development (Liang et al., 2021; Boorboori and Zhang, 2022). They are backed by the fact that soil nutrient concentrations and plant biomass positively correlate after *P. indica* inoculation (**Figure 4**).

The accumulation and transportation of photosynthates and dry matter within plant leaves are closely related. It has been established that salinity stress not only diminishes the production of photosynthetic pigments but also inhibits plant growth and development (Peng et al., 2012). This is explained by the build-up of reactive oxygen species in stressed plants, which has a detrimental effect on the growth and metabolism of plants (Sharma et al., 2012). The results of this study showed a significant increase in net photosynthetic and transpiration rates of plants under the co-application of biochar and *P. indica*, which suggests that biochar and *P. indica* can reduce the negative effects of salinity on plants by improving their biological growth and photosynthetic activity (Thomas et al., 2013). The observation is primarily ascribed to the increased antioxidant enzyme activity induced by *P. indica*, thereby enhancing photosynthetic pigment synthesis (Shukla et al., 2008), improving the photosynthetic rate of plants, and alleviating salinity stress by maintaining tissue osmotic pressure (Zhao, 2011; Zarea et al., 2012). Reports indicate that the application of biochar enhances chlorophyll synthesis and maintains leaf water content, while concurrently reducing the accumulation of proline, H_2_O_2_, and malondialdehyde (Huang et al., 2023).

The variation in the effects of the various forms of biochar on intercellular CO_2_ concentration and stomatal conductance could be associated with the variations in the properties of biochar materials that, in turn, affected the inter-root microbial community and population (Wang et al., 2024). Prevention of water loss from plant leaves through transpiration under limited water availability conditions, such as saline soils, is essential and can be achieved by stomatal closure or reduction of leaf area (Parmar et al., 2013; Lin et al., 2017). Likewise, Usman et al. (2016) found that biochar application improved gas exchange parameters in tomato leaves, promoted nutrient uptake and phytohormone regulation, and improved the quality of tomatoes irrigated with brackish water.

Leaf chlorophyll content increased significantly with an increase in *P. indica* dosage under the combined application of biochar and *P. indica*. The elevated chlorophyll content observed in this study was paralleled by an increased net photosynthetic rate and enhanced plant growth consistent with findings from a previous study (Zhang et al., 2018). The combined application of biochar and *P. indica* protects plants against salinity stress, enhances the photosynthetic capacity of plants, and accelerates dry matter accumulation, thereby encouraging plant growth (Gururani et al., 2015).

## 5 Conclusions

The synergistic application of biochar and *P. indica* has been shown to enhance the growth of sorghum–sudangrass hybrids by optimizing the rhizosphere micro-ecological environment and augmenting plant photosynthetic efficiency. Specifically, the co-application of biochar and *P. indica* resulted in a reduction of electrical conductivity and pH levels, facilitated the absorption of water and nutrients, and notably increased chlorophyll content, net photosynthetic rate, and transpiration rate. These enhancements collectively led to a boost in the biomass of the sorghum–sudangrass hybrids. Notably, bamboo biochar exhibited superior performance over corn stover biochar in elevating the transpiration rate and net photosynthetic rate. Moreover, when combined with a high dosage of *P. indica* (120 ml/pot), bamboo biochar demonstrated enhanced efficacy in improving soil quality and fostering the growth of sorghum–sudangrass hybrids. Consequently, the integrated application of biochar and *P. indica* represents a viable strategy for ameliorating the quality of coastal saline–alkali soils and ensuring the normal growth of plants in saline environments. Moving forward, greater emphasis should be placed on research pertaining to the optimal dosages and application methods of biochar and microbial agents, with the aim of achieving enhanced outcomes in soil quality improvement and plant growth promotion.

## Data Availability

The original contributions presented in the study are included in the article/supplementary material. Further inquiries can be directed to the corresponding author. Requests to access these datasets should be directed to ZD, dongzhi@sdau.edu.cn.
